# Acylcarnitine Profiles in Plasma and Tissues of Hyperglycemic NZO Mice Correlate with Metabolite Changes of Human Diabetes

**DOI:** 10.1155/2018/1864865

**Published:** 2018-04-26

**Authors:** Anna Weiser, Pieter Giesbertz, Hannelore Daniel, Britta Spanier

**Affiliations:** Nutrition Physiology, Technische Universität München, Gregor-Mendel-Str. 2, 85350 Freising, Germany

## Abstract

The New Zealand obese (NZO) mouse is a polygenic model for obesity and diabetes with obese females and obese, diabetes-prone males, used to study traits of the metabolic syndrome like type 2 diabetes mellitus (T2DM), obesity, and dyslipidaemia. By using LC-MS/MS, we here examine the suitability of this model to mirror tissue-specific changes in acylcarnitine (AC) and amino acid (AA) species preceding T2DM which may reflect patterns investigated in human metabolism. We observed high concentrations of fatty acid-derived ACs in 11 female mice, high abundance of branched-chain amino acid- (BCAA-) derived ACs in 6 male mice, and slight increases in BCAA-derived ACs in the remaining 6 males. Principal component analysis (PCA) including all ACs and AAs confirmed our hypothesis especially in plasma samples by clustering females, males with high BCAA-derived ACs, and males with slight increases in BCAA-derived ACs. Concentrations of insulin, blood glucose, NEFAs, and triacylglycerols (TAGs) further supported the hypothesis of high BCAA-derived ACs being able to mirror the onset of diabetic traits in male individuals. In conclusion, alterations in AC and AA profiles overlap with observations from human studies indicating the suitability of NZO mice to study metabolic changes preceding human T2DM.

## 1. Introduction

With the generation of the first New Zealand obese (NZO) mouse strain in 1948, a polygenic mouse model entered the diabetes research field which in comparison to monogenic db/db and ob/ob mice has been much less studied and for which “omics studies” are rare [1]. Previously, we studied the monogenic db/db and ob/ob mouse models [2] as well as a mouse model of diet-induced obesity (DIO) [3]. These served as comparative models to elicit and evaluate strengths and weaknesses of the inbred NZO mouse strain to study obesity-induced type 2 diabetes mellitus (T2DM). Although these models all develop obesity, they differ in their susceptibility to developing diabetes. Whereas ob/ob mice develop a strong obesity phenotype, they display an only mild and temporal hyperglycemia associated with pancreatic hypertrophy and hyperplasia. In contrast, db/db mice and male NZO mice suffer from brittle *β*-cells leading to a severe hyperglycemia and the progression of T2DM [2, 4]. Approximately 50% of the male NZO mice exert T2DM accompanied with a mature hyperinsulinemia and a subsequent *β*-cell destruction while females remain nondiabetic due to protective effects of oestrogen [5–7]. It was shown that oestrogen decreases hyperphagia via hypothalamic stimuli and furthermore leads to enhanced glucose tolerance, higher insulin sensitivity, and an improved *β*-cell integrity [8].

Differences between the obesity models can be found as well for their metabolite profiles. Whereas both the monogenic ob/ob and db/db mice display elevated concentrations of branched-chain amino acids (BCAA) as well as BCAA-derived metabolites as robust markers for human T2DM [9], DIO mice do not display this characteristic diabetic fingerprint [3]. Investigation of plasma acylcarnitine (AC) and amino acid (AA) profiles in the DIO C57BL/6 mouse model before and 10 weeks after a high-fat feeding imparted weak predictive value of plasma AC and plasma AA as markers to separate obese insulin-sensitive mice from obese mice with impaired insulin homeostasis [10].

So far, a comprehensive metabolite profiling of the NZO mouse model has not been established. Here, we used targeted metabolite profiling and studied the results of 43 ACs and 35 AAs and their derivatives derived from plasma, liver, kidney, heart muscle, skeletal muscle, and adipose tissue to decipher tissue-specific underlying metabolic alterations that precede T2DM in the NZO strain.

## 2. Material and Methods

### 2.1. Animals

The inbred mouse line NZO/HIBomDife was kindly provided from DIfE Institute (Nuthetal, Germany). The strain was originally received from Bomholtgard (Ry, Denmark) and kept as a separate mouse line at DIfE since more than 15 years. The mice were bred in our animal facility, and 12 age-matched males and 11 females were obtained for this study. After 3 weeks of weaning, the mice were subjected to a standard chow diet (ssniff V1534-0 R/M-H) for 5 weeks followed by a chemically defined and controlled diet (CCD; ssniff E15000-04 EF R/M Kontrolle) for 12 weeks. All mice had ad libitum access to clean water and food and were kept in an open mouse facility during the entire study. At 20 weeks of age, body weight and blood glucose were measured in a nonfasted state followed by cervical dislocation under anaesthesia using isoflurane (Baxter, Unterschleißheim, Germany). All animal procedures were in accordance with the Principles of Laboratory Care and approved by the Veterinary Inspection Services.

### 2.2. Plasma and Tissue Collection

Blood samples were obtained via retroorbital puncture and transferred into EDTA-coated tubes prior to centrifugation at 1.200*g* and 4°C for 10 min to extract plasma. Mice were rapidly dissected, and tissues comprising kidney, liver, heart muscle, skeletal muscle, and adipose tissue were transferred into respective tubes. Plasma and tissues were snap-frozen in liquid nitrogen and stored at −80°C until analysis. For subsequent sample preparation, tissues were ground under constant use of liquid nitrogen to ensure intactness of metabolites.

### 2.3. Metabolite Profiling via LC-MS/MS

AC and AA profiles were obtained from plasma, skeletal muscle, liver, kidney, heart muscle, and adipose tissues, using a triple quadrupole QTRAP 5500 tandem mass spectrometer (AB Sciex, Framingham, MA, USA) coupled to an Agilent 1260 Infinity Binary HPLC system (Agilent Technologies, Waldbronn, Germany) and an HTC PAL autosampler (CTC Analytics, Zwingen, Switzerland). Plasma and tissues were extracted with methanol and extracts were derivatized to their butyl esters as described previously [11, 12]. The butylated samples additionally contained 14 stable isotope-labelled ACs and 16 deuterated AAs as internal standards. Chromatographic separation was achieved on a reversed-phase Zorbax Eclipse XDB-C18 column (Agilent Technologies, Waldbronn, Germany).

### 2.4. Analysis of NEFA, Urea, Insulin, and TAG

Quantification of plasma insulin concentrations was performed using the ultrasensitive mouse insulin ELISA kit from Crystal Chem (IL, USA). Nonesterified fatty acids (NEFA) were measured by using the NEFA-HR(2) ACS-ACOD Kit from Wako Chemicals (Neuss, Germany). The concentration of plasma triacylglycerols (TAGs) was determined using a Triglyceride LiquiColor Kit from HUMAN Diagnostics (Wiesbaden, Germany), and urea concentrations in the plasma samples were specified with the Urea Liquicolor Kit from HUMAN Diagnostics.

### 2.5. Data Analysis

Area ratios of the AC peaks in the chromatogram were calculated using the Analyst 1.5.1 software and converted into concentrations in *μ*mol/l. Multivariate and exploratory data analysis as well as statistical testing were accomplished in R. To reveal the optimal number of clusters, a scree plot followed by hierarchical clustering of principle components (HCPC) and a subsequent *k*-means consolidation were applied on samples from plasma and each tissue using the R-package FactoMineR. Metabolites were used to further describe the dimensions of the HCPC. Significant metabolites for each cluster were listed, and respective category means were compared to the overall means using a Student's *t*-test. Obtained *p* values were transformed to a normal quantile using the *v*-test transformation by Lebart et al. [13, 14].

Differences among gendered groups (female, male, and diabetic male NZO mice) regarding plasma parameters like blood glucose, insulin, TAG, NEFA, and urea were statistically investigated using ANOVA based on a previous data transformation with the negative reciprocals of the data to achieve normal distribution. For differences between the three groups concerning ACs and AAs in plasma and tissues, a Kruskal-Wallis test was applied to detect significant values. Results were considered as significant at an alpha level of 0.05.

## 3. Results

**Figure 1:**
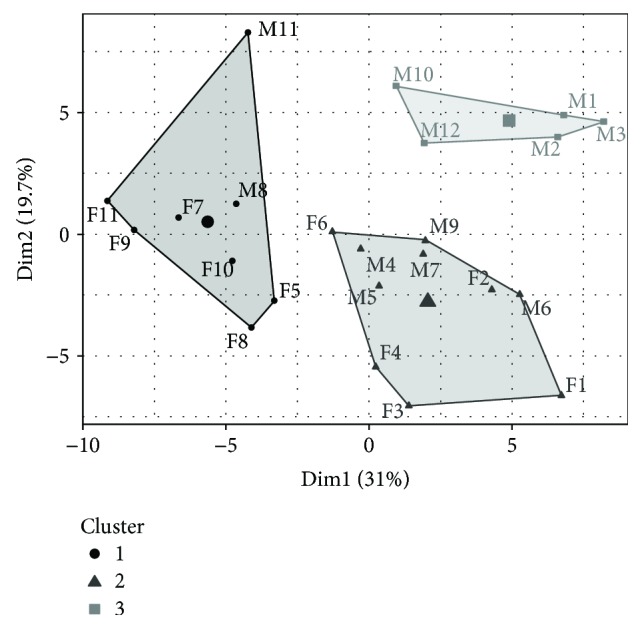
Plasma-derived cluster plot of 12 male and 11 female NZO mice to identify groups. HCPC provided three distinct clusters. Clusters 1 and 2 contain female and male NZO mice whereas cluster 3 includes solely male NZO mice. The clustering is based on the input of 78 measured metabolites comprising 43 ACs and 35 AAs. Gender is marked with M (male) and F (female).

HCPC derived from plasma samples separated mice into three distinct clusters. One cluster consisted solely of male animals (cluster 3) while the remaining clusters comprised both genders (Figure 1). Significantly differing metabolite concentrations between the clusters were computed and are listed in Supplemental Table A.1. Cluster 1 was marked by elevated even-numbered long-chain fatty acid- (LCFA-) derived ACs like octadecenyl-carnitine (C18:1), octadecadienyl-carnitine (C18:2), hexadecenyl-carnitine (C16:1), and octadecanoyl-carnitine (C18) and very low abundant BCAAs and BCAA-derived ACs. Cluster 2 was most distinct by high concentrations of essential AAs like threonine (Thr), lysine (Lys), tryptophan (Trp), and methionine (Met). Medium-chain fatty acid (MCFA) and LCFA-derived ACs were strongly negatively linked to cluster 2. For cluster 3, especially high amounts of odd-numbered fatty acid-derived ACs like heptanoyl-carnitine (C7) and pentanoyl-carnitine (C5) as well as BCAA-derived ACs like 2-methylbutyryl-carnitine (2-M-C4) and isovaleryl-carnitine (3-M-C4) showed highly significant results while serine, glycine, alpha-aminoadipic acid (AADP), pentadecenoyl-carnitine (C15), and cystine (Cys-Cys) showed negative associations with cluster 3.

**Figure 2:**
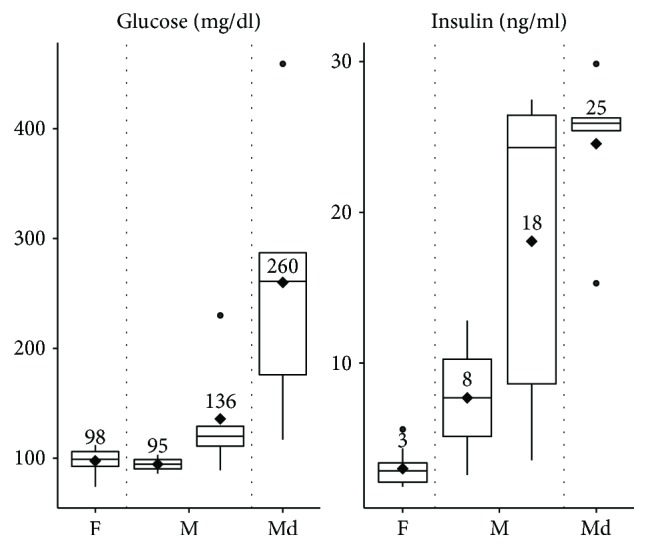
Identification of nondiabetic, prediabetic, and diabetic subjects based on blood glucose and plasma insulin concentrations. Plasma glucose and insulin levels were separately analysed based on the HCPC-derived clusters for nondiabetic females (F), non- and prediabetic male mice from clusters 1 and 2 (M), and diabetic male mice from cluster 3 (Md). Rhombuses depict mean values.

From the three clusters that were found based on metabolite profiling in plasma, we examined variations in blood glucose and insulin concentrations for both genders (Figure 2). As to be expected, females did not show diabetic marks due to the protective effect of oestrogen. For that reason, we focused on the diabetes-prone male mice. When considering all male animals in the three clusters, males in cluster 3 showed significantly increased blood glucose values with 260 ± 116 mg/dl (mean ± SD) as well as elevated plasma insulin concentrations with 25 ± 5 ng/ml compared to males in clusters 1 and 2 (Figure 2). Males from cluster 1 showed the lowest blood glucose concentrations with 95 ± 9 mg/dl, comparable to the levels of females in this study with 98 ± 11 mg/dl. However, males from cluster 1 exerted higher insulin concentrations of 8 ± 5 ng/ml compared to females with 3 ± 1 ng/ml. Males from cluster 2 showed intermediate levels of glucose with 136 ± 49 mg/dl and insulin concentrations of 18 ± 10 ng/ml which is consistent with an intermediate condition of hyperglycemia and hyperinsulinemia between nondiabetic and diabetic animals from clusters 1 and 3, respectively.

To reduce the confounding influence of gender specificity, females were kept as one group (F). Males from clusters 1 and 2 were aligned to group M including nondiabetic and prediabetic male mice to increase the number of mice per cluster to gain statistical power as prerequisite for statistical testing. The drawback of increasing group size is the loss of the distinction between healthy and prediabetic male mice which would complete the line-up of dynamic changes in metabolite patterns allowing to characterize nondiabetic, prediabetic, and diabetic mice. Males from cluster 3 consistently exerted diabetic blood glucose concentrations and were denoted as male diabetic (Md).

**Figure 3:**
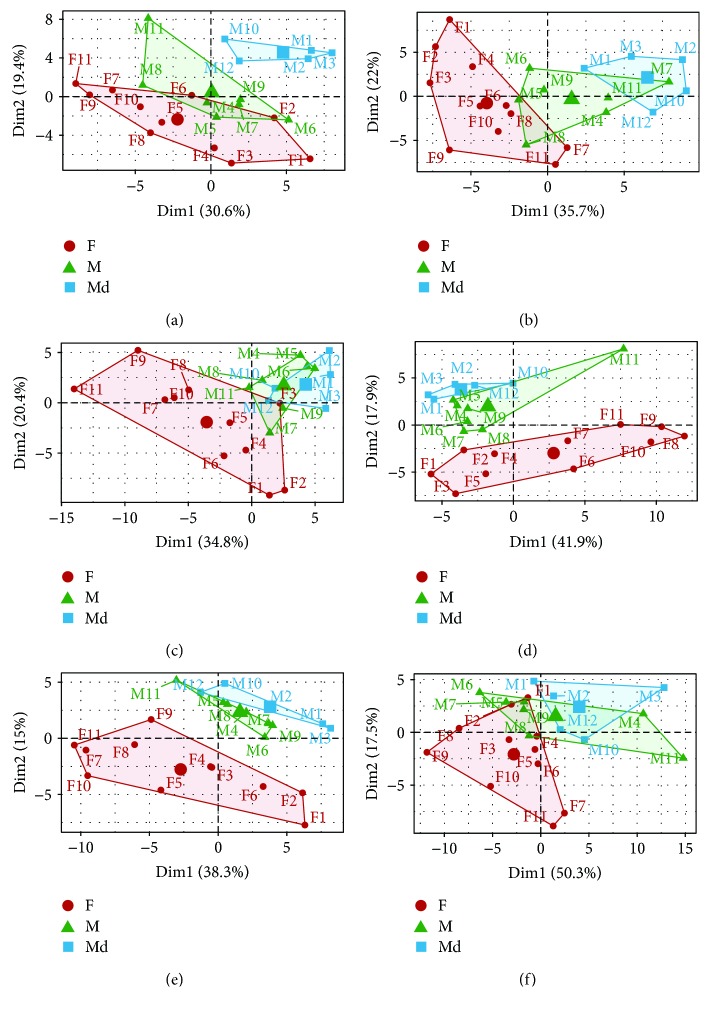
PCA to characterize plasma- and tissue-specific metabolite profiles in 12 male and 11 female NZO mice. (a) Plasma; (b) adipose tissue; (c) liver; (d) muscle; (e) heart; (f) kidney. PCAs comprise data of 43 ACs and 35 AAs. Groups are coloured according to female (F), male (M), and male diabetic (Md) mice to examine differences and resemblances among plasma and tissue metabolite profiles. Gender is marked with F (female) and M (male).

Plasma- and tissue-specific PCAs showed metabolite-based patterns of similarity among the three mouse groups (Figure 3). In plasma, the metabolite structure of the Md group (Figure 3(a)) is most distinct from M and F compared to the tissue-derived PCAs (Figures 3(b)–3(f)). Next to plasma, adipose tissue is best suited to separate the two male groups (Figures 3(a) and 3(b)). Opposed to this, hepatic and renal tissues depict mainly overlapping features of the M and Md groups (Figures 3(c) and 3(f)).

In the following, only data from the two male groups M and Md will be compared regarding changes in metabolite profiles associated to hyperglycemia. Concentrations that were significantly different between M and Md were depicted as fold changes in Table 1. The derived significant metabolites were coincident with characterizing plasma metabolites for cluster 3 derived from the HCPC (Table A.1). Individuals from groups Md and M were further tested for significant differences in kidney, liver, heart muscle, skeletal muscle, and adipose tissue. Significantly changed hepatic metabolites between Md and M included heptanoyl-carnitine (C7), nonanoyl-carnitine (C9), and 2-methylbutyryl-carnitine (2-M-C4) and appeared to be most akin to those found in plasma opposed to significant metabolites in other tissues (Table 1).

Further phenotypic characteristics of M and Md regarding plasma-derived traits like TAGs, NEFAs, urea, insulin, and blood glucose were examined. However, except for blood glucose concentrations, none of the remaining traits showed a significant difference between M and Md (Table A.2).

**Figure 4:**
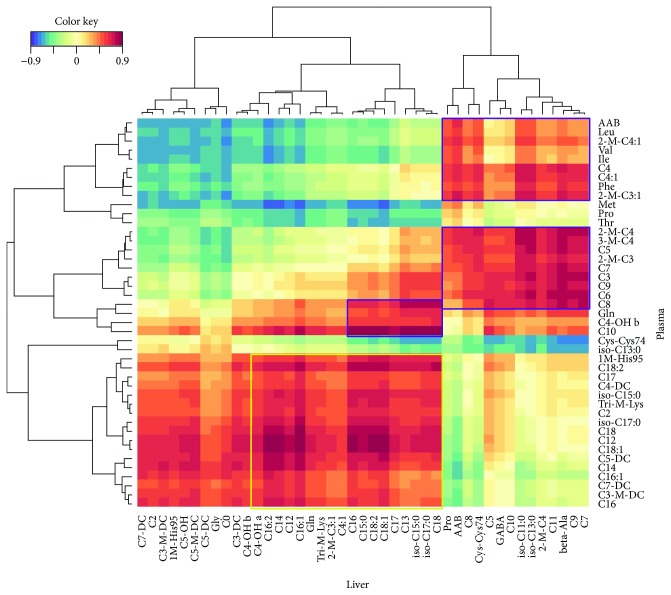
Heat map of hierarchical clustering of plasma and liver metabolites from diabetic (Md) and non-/prediabetic (M) male NZO mice. Highly correlating metabolites from plasma and liver are marked with coloured frames, while purple frames comprise mainly BCAA-derived and MCFA-derived ACs while yellow-marked frames comprise mainly LCFA-derived ACs. The threshold for the absolute correlation was set to at least 0.6. The colour scale reflects the distance score between each metabolite pair, with dark red indicating strong positively correlated pairs of metabolites and blue indicating strong negative correlations.

Since most significant metabolites were found in plasma and liver, a more detailed analysis of the relations among respective metabolites was performed. For this, relations between plasma and liver metabolites with absolute correlation coefficients of at least 0.6 for group M and Md were depicted (Figure 4).

Squares with highly correlated metabolites between liver and plasma were marked with coloured frames. Metabolites within the purple frame are dominated by plasma BCAAs and their downstream derived ACs as well as by MCFA-derived ACs which mainly correlated with MCFA-derived ACs in liver tissue. Plasma BCAA-derived and MCFA-derived ACs with significant correlations with hepatic MCFA are 2M-C4, 3M-C4, C4, C4:1, 2M-C3, 2M-C3:1, C3, C5, C6, C7, C8, C9, and C10. Very strong correlations exist between plasma-derived C10 and several LCFA-derived ACs from liver tissue. The yellow frame marks a group of hepatic LCFA-derived ACs which correlate mainly with LCFA-derived ACs from plasma like C18:1, C18:2, C16, C16:1, C16:2, C14, and C15. The correlations do not allow to conclude biochemical relations among liver and plasma; however, it delivers a good overview about plasma metabolites that were previously detected as significant and their closest correlating metabolites from liver tissue and vice versa. This may be useful as a preselection to identify metabolites that dominate the pattern of dysregulated fatty acid oxidation leading to metabolic perturbations like the onset of T2DM.

## 4. Discussion

### 4.1. NZO Mice Display Obesity-Induced Insulin Resistance with the Potential to Develop Diabetes

Among the most widely investigated T2DM mouse models are the monogenic ob/ob and db/db mice as well as models of high-fat DIO and insulin resistance. These models differ in their susceptibility and their speed in which they progress from an obese insulin-resistant towards an insulin-deficient type 2 diabetic state, depending on the stability of their *β*-cells. Moreover, we and others observed distinct metabolic alterations between the respective models. BCAAs, prominent marker metabolites of human T2DM, were reported as increased in the monogenic ob/ob and db/db mice [2], but these changes could not be confirmed in DIO mice [3]. In the present study, we examined NZO mice as a polygenic model of obesity-induced insulin resistance and T2DM. This model has been used in quantitative trait locus analysis to discover risk alleles for the development of T2DM [15, 16], but is rather scarcely studied within the context of metabolite alterations related to obesity-induced T2DM. Here, we aimed to explore metabolite alterations in the NZO model to determine to what extent these mice can reproduce respective metabolic conditions in human. Using a targeted LC-MS/MS analysis, we explored changes in AA and AC concentrations, which include BCAAs and BCAA-derived metabolites, described as prominent diabetes markers.

The preceding metabolite profiling in monogenic ob/ob and db/db mice [2] and mice fed a high-fat diet [3] allowed to perform a comparison of metabolite profiles in the NZO mice studied here with our previous measurements.

In our study, female mice remained normoglycemic, which is in line with previous literature stating protective effects of oestrogen [1, 6]. We furthermore observed a large variation in the development and severity of hyperglycemia within the male group since numbers for age and fasted blood glucose concentrations at the onset of T2DM vary among the studies and make it difficult to define the beginning of diabetic perturbations by distinct biomarkers [2, 17, 18]. Previous studies describe that only around 50% of male animals progress to a state of T2DM [1].

One reason for this is the progression of obesity in NZO mice which determines the development of diabetes-like metabolic circumstances [1]. Dependent on the fed diet and the individual progression of gaining weight, overt diabetes-like conditions can appear already in 5-week-old NZO mice with blood glucose levels higher than 300 mg/dl [19]. Besides a large interindividual genetic variation in obesity genes determining the progression speed towards obesity, variation in a large number of other genes determines the progression of individual mice towards obesity-associated diabetes [20]. The comparison of our results derived from db/db and NZO mice showed a more progressive severity of T2DM in db/db opposed to NZO mice when comparing the average blood glucose levels at an age of 20 weeks. However, on an individual basis, it is important to keep in mind that the development of T2DM in NZO is a more dynamic procedure as compared to the db/db model.

### 4.2. Hyperglycemic Male NZO Mice Show Increased Plasma Levels of BCAA-Derived and MCFA-Derived ACs, but Not of BCAAs

Using the targeted metabolite profiles from plasma, we defined three distinct animal clusters, one consisting only of male animals (Md) showing increased blood glucose levels as compared to other males (M) distributed within the two remaining clusters. Males from group M comprised non- and prediabetic male mice to increase group size for statistical testing. This, however, represents a limitation of our study since it does not allow to display a full set of metabolite patterns covering nondiabetic, prediabetic, and diabetic male NZO mice. Additional plasma metabolites like urea, insulin, and NEFAs did not show significant changes between the male groups. When comparing metabolite concentrations in plasma between hyperglycemic Md and normoglycemic M males, it is striking to find significant increases in BCAA derived and short- and MCFA-derived ACs but no significant changes in BCAAs especially since the HCPC output stated BCAA as significant metabolites for cluster 3 containing diabetic male mice. An explanation for this conflicting result is the difference concerning the structure of the groups. For cluster analysis, one male and two mixed clusters were examined while for the statistical testing only the two male groups M and Md were considered. The lacking significant difference of BCAA between the Md and M groups might also be influenced by the fact that M comprises prediabetic mice shifting the metabolite pattern closer to the Md group. The significant difference among the male groups regarding the BCAA-derived ACs suggests a dysregulation more downstream of mitochondrial BCAA breakdown. A likely involvement of short-chain 3-hydroxyacyl-CoA dehydrogenase (SCHAD) might be expected, as its according gene (Hadh) was previously identified as an obesity gene via quantitative trait locus analysis in NZO mice [21]. Furthermore, SCHAD has been shown to impact thermogenesis and nutrient-dependent insulin secretion, characterizing knockout mice with significantly lowered blood glucose levels and increased insulin concentrations. It is likely that SCHAD exerts an important regulatory impact on the usage of ACs as energy source. In our study, MCFA-derived ACs up to iso-C13 were significantly characteristic for hepatic tissue of Md mice. MCFA-derived ACs are potential indicators to reflect an incomplete fatty acid oxidation imparting a progressive dysregulation of lipid metabolism [22]. At the age of 20 weeks, metabolite profiles of male mice were not dominated by LCFAs; however, MCFAs have been revealed to be characterizing for the progression towards T2DM. For the dynamic NZO model subjected to a CCD, the effects of an impaired *β*-oxidation are not as distinct as they might evolve under a high-fat diet and therefore are only expected to evolve over time with the progression of obesity-induced T2DM. ACs derived from C12 to C14 fatty acids are associated with the inflammatory transcription factor NF*κ*B triggering a low chronic inflammation that drives obesity-associated insulin resistance [23]. Except for iso-C13 in liver tissue, there were no significant differences between M and Md for C12, C13, and C14. In comparison to female mice, all male mice exerted higher C12 to C14 metabolites in adipose tissue whereas other tissues did not show such a distinct separation between genders (data not shown).

It is of note that a comparison of plasma AA profiles between NZO and New Zealand black (NZB) mice did show increased levels of BCAAs in the NZO mice [24]. Important here is that NZB mice are lean. This is in line with reports showing that BCAAs already increase in an early obesity-induced insulin-resistant state before the onset of diabetes [25]. However, a further increase in BCAAs in an obese diabetic as compared to an obese nondiabetic state thus seems unlikely based on our results.

### 4.3. Tissue Metabolite Profiles Suggest a Role for the Liver in the Generation of Odd-Numbered Carbon Chains

The influence of plasma and tissue on the allocation of metabolites including AAs and ACs is still poorly understood. While most data sets in literature are coming from plasma, it is important to note that this matrix is the result of the influx and efflux of AAs and ACs from various tissues without knowing which tissue exhibits strong or weak influence on the metabolite profile [26]. In a similar approach to our previous study with ob/ob and db/db mice [2], metabolite profiling of individual tissues from NZO mice was performed and showed most pronounced changes in liver. This is in contrast to the db/db mice in which a correlation between plasma and adipose tissue was reported. However, earlier Shin et al. stated an impact of hepatic BCAA metabolism on the BCAA concentration in plasma [27]. Brain insulin lowers circulating BCAA levels by inducing hepatic BCAA catabolism [27]. Increases in hepatic concentrations of various odd-numbered AC species can be observed, including heptanoyl-carnitine (C7), nonanoyl-carnitine (C9), and undecanoyl-carnitine (C11), as well as isomeric forms of undecanoyl-carnitine (iso-C11) and tridecanoyl-carnitine (iso-C13). The exact origin for these odd-numbered carbon chains remains unknown, and various metabolic routes of formation have been described. Peroxisomal processes of fatty acid alpha oxidation are known to generate odd-numbered carbon chains by chain-shortening fatty acids by one single carbon atom. This is however thought to be a minor process and seems important in catabolic situations [28]. More recently, a study in adipocytes shows that it is principally possible to generate longer odd-numbered acyl chains via elongation of BCAA-derived carbon chains [29].

## 5. Conclusion

In summary, our study has examined the NZO mouse model by investigating AC and AA species in plasma, liver, kidney, skeletal muscle, heart muscle and adipose tissue to provide detailed and comprehensive fingerprints of different states in the development of T2DM in male mice—from non- to prediabetic to severely diabetic states. Thereby, metabolites derived from BCAAs as well as MCFA derived ACs turned out to be characteristic for diabetic perturbations in male NZO mice. This is coinciding with studies conducted in human trials and highlights the benefits of the polygenic NZO mouse model in mirroring human T2DM conditions for further investigations.

## Figures and Tables

**Table 1:** Metabolic alterations between the group of diabetic male (Md) and the group of non-/prediabetic male (M) NZO mice in plasma, tissues, and phenotypic traits.

Metabolite	Abbreviation	Fold changes Md : M					
		Plasma	Liver	Adipose tissue	Muscle	Kidney	Heart
Heptanoyl-C	C7	**3.21** ^∗^	**3.98** ^†^	1.33	1.25	1.18	1.51
Pentanoyl-C	C5	**2.31** ^∗^	**1.87** ^ǂ^	1.86	**1.55** ^∗^	1.40	**1.73** ^ǂ^
Glucose	Glc	**2.10** ^†^	2.10	2.10	2.10	2.10	2.10
Crotonyl-C	C4:1	**2.07** ^∗^	0.92	1.56	1.71	3.03	1.29
Hexanoyl-C	C6	**2.04** ^∗^	1.89	1.10	1.13	0.98	1.52
2-Methylbutyryl-C	2-M-C4	**2.04** ^∗^	**1.73** ^∗^	1.28	1.41	1.47	**1.61** ^∗^
Isobutyryl-C	2-M-C3	**1.99** ^∗^	0.81	1.25	1.34	0.97	0.65
Isovaleryl-C	3-M-C4	**1.97** ^∗^	0.64	1.16	1.16	1.52	**1.44** ^ǂ^
Nonanoyl-C	C9	**1.90** ^†^	5.35^†^	1.24	0.76	0.99	1.12
Propionyl-C	C3	**1.88** ^∗^	1.17	1.28	1.05	1.30	1.30
Methacrylyl-C	2-M-C3:1	**1.88** ^ǂ^	0.90	**1.45** ^∗^	**2.63** ^ǂ^	3.03	1.60
Butyryl-C	C4	1.74	0.87	1.16	**1.44** ^ǂ^	1.14	1.14
3-Methylcrotonyl-C	3-M-C4:1	1.73	0.92	**<LOQ** ^¶^	1.28	1.33	**1.60** ^ǂ^
Octanoyl-C	C8	1.66	**3.13** ^∗^	0.95	0.84	0.94	1.15
Tiglyl-C	2-M-C4:1	1.52	1.17	1.42	1.39	1.69	**1.45** ^∗^
Isoundecanoyl-C	iso-C11:0	1.45	**5.86** ^†^	1.28	0.88	1.44	2.29
*α*-Aminobutyrate	AAB	1.34	**1.66** ^ǂ^	1.32	1.15	1.48	1.39
Glutamine	Gln	**1.31** ^∗^	0.98	**1.21** ^ǂ^	0.90	1.13	0.99
Hydroxybutyryl-C-b	C4-OH b	1.31	0.75	1.27	**1.77** ^∗^	1.07	1.46
Isoleucine	Ile	1.30	1.01	**1.38** ^∗^	1.10	1.11	1.09
Phenylalanine	Phe	**1.27** ^ǂ^	1.19	1.18	0.97	1.13	1.12
*β*-Alanine	*β*-Ala	1.15	**2.07** ^ǂ^	1.37	0.94	1.29	1.16
*γ*-Aminobutyric acid	GABA	1.08	1.38	4.02	1.11	**1.54** ^∗^	1.20
Tryptophan	Trp	1.05	3.83	1.34	1.16	**<LOQ** ^¶^	**1.47** ^ǂ^
Hydroxybutyryl-C-a	C4-OH a	1.01	0.77	1.29	**1.80** ^†^	0.97	1.00
Arginine	Arg	0.91	0.85	1.04	**0.57** ^ǂ^	0.84	0.82
3-Methyl-Histidine	3M-His	0.90	**1.29** ^ǂ^	1.28	0.98	1.23	1.02
Methylglutaryl-C	C5-M-DC	0.62	0.75	**1.34** ^ǂ^	0.97	0.74	1.12
Cystine	Cys-Cys	**0.46** ^∗^	**2.44** ^ǂ^	1.63	1.20	1.48	1.85
Undecanoyl-C	C11	0.19	**3.18** ^†^	1.69	0.91	1.09	0.75
Isotridecanoyl-C	iso-C13:0	0.14	**4.33** ^∗^	1.01	0.99	1.41	1.18

Differences in plasma- and tissue-derived ACs and AAs between diabetic male NZO mice (Md) and non-/prediabetic male mice (M). Metabolites with *p* values above 0.05 are shown since the study aims for a holistic approach to define metabolite profiles for diabetic conditions rather than identifying single markers for T2DM. Results were considered as significant at a threshold of α < 0.05. LOQ = limit of quantification; C = carnitine. ^¶^<LOQ; ^†^0.001 < *p* < 0.01; ^∗^0.01 < *p* < 0.05; and ^ǂ^0.05 < *p* < 0.1.

## Abbreviations

AA: Amino acid
AADP: Alpha-aminoadipic acid
AC: Acylcarnitine
BCAA: Branched-chain amino acid
C: Carnitine
CCD: Chemically defined and controlled diet
DIO: Diet-induced obesity
EDTA: Ethylenediaminetetraacetic acid
Glc: Glucose
HCPC: Hierarchical clustering of principal components
HOMA-IR: Homeostatic model assessment-insulin resistance
LCFA: Long-chain fatty acid
MCFA: Medium-chain fatty acid
Md: Male diabetic
NEFA: Nonesterified fatty acid
NZB: New Zealand black
NZO: New Zealand obese
PCA: Principal component analysis
SCHAD: Short-chain 3-hydroxyacyl-CoA dehydrogenase
T2DM: Type 2 diabetes mellitus
TAG: Triacylglycerol.

## Supplementary Materials

Supplementary 1 Supplemental Table A.1: descending order from highly positively related to highly negatively related plasma metabolites for clusters 1, 2, and 3 based on Figure 1.

Supplementary 2 Supplemental Table A.2: ANOVA test results and fold changes from plasma-derived phenotypic traits including blood glucose, insulin, TAG, NEFA, and urea to reveal differences among diabetic male NZO mice (Md) and non-/prediabetic male NZO mice (M).
