# Supplementary material for: Acylcarnitine Profiles in Plasma and Tissues of Hyperglycemic NZO Mice Correlate with Metabolite Changes of Human Diabetes
Source: J Diabetes Res. 2018 Apr 26;2018:1864865. doi: 10.1155/2018/1864865 (PMC5944288; doi:10.1155/2018/1864865)
Supplement: Supplementary 1 — Supplemental Table A.1: descending order from highly positively related to highly negatively related plasma metabolites for clusters 1, 2, and 3 based on Figure 1. [file 1864865.f1.docx]

Supplemental Table A.1. Descending order from highly positive related to highly negative related metabolites for each cluster.

| **Cluster 1** | |  |  | **Cluster 2** | |  |  | **Cluster 3** | |  |
| --- | --- | --- | --- | --- | --- | --- | --- | --- | --- | --- |
| **Metabolite** | **v.test** | **p.value** |  | **Metabolite** | **v.test** | **p.value** |  | **Metabolite** | **v.test** | **p.value** |
| C18:1 | 3,76 | 0,0002 |  | Thr | 2,82 | 0,0049 |  | C7 | 3,95 | 0,0001 |
| C16:1 | 3,71 | 0,0002 |  | Lys | 2,73 | 0,0064 |  | 2-M-C4 | 3,83 | 0,0001 |
| C14 | 3,61 | 0,0003 |  | Pro | 2,69 | 0,0071 |  | 3-M-C4 | 3,80 | 0,0001 |
| C18:2 | 3,51 | 0,0005 |  | Orn | 2,53 | 0,0114 |  | C5 | 3,78 | 0,0002 |
| C3-M-DC | 3,41 | 0,0006 |  | Trp | 2,16 | 0,0308 |  | 2-M-C3:1 | 3,68 | 0,0002 |
| iso-C17:0 | 3,41 | 0,0007 |  | Met | 2,11 | 0,0346 |  | 2-M-C3 | 3,67 | 0,0002 |
| C18 | 3,19 | 0,0014 |  | Ala | 2,10 | 0,0359 |  | C4:1 | 3,65 | 0,0003 |
| C6-DC | 3,18 | 0,0015 |  | Asn,144,2 | 1,97 | 0,0486 |  | C4 | 3,59 | 0,0003 |
| C16 | 3,18 | 0,0015 |  | Cys-Cys74 | 1,97 | 0,0489 |  | C6 | 3,40 | 0,0007 |
| C12 | 3,14 | 0,0017 |  | OH-Pro | -2,00 | 0,0459 |  | C9 | 3,14 | 0,0017 |
| Gly | 2,92 | 0,0035 |  | C13 | -2,05 | 0,0402 |  | 3-M-C4:1 | 3,11 | 0,0019 |
| iso-C15:0 | 2,82 | 0,0048 |  | C14 | -2,06 | 0,0392 |  | C3 | 3,09 | 0,0020 |
| Butyrobetaine | 2,71 | 0,0067 |  | C18:1 | -2,30 | 0,0217 |  | Phe | 2,99 | 0,0028 |
| Carnosine.110 | 2,62 | 0,0089 |  | C6-DC | -2,34 | 0,0192 |  | 2-M-C4:1 | 2,80 | 0,0052 |
| C13 | 2,56 | 0,0105 |  | C10 | -2,34 | 0,0191 |  | OH-Pro | 2,67 | 0,0076 |
| C10 | 2,47 | 0,0134 |  | Trimethyllysine | -2,35 | 0,0187 |  | Glu | 2,48 | 0,0130 |
| C2 | 2,40 | 0,0162 |  | Gln | -2,38 | 0,0172 |  | C5-OH | 2,46 | 0,0139 |
| C4-OH,b | 2,23 | 0,0258 |  | C12 | -2,38 | 0,0172 |  | Leu | 2,41 | 0,0158 |
| C5-DC | 2,21 | 0,0269 |  | C18 | -2,38 | 0,0172 |  | Val | 2,35 | 0,0186 |
| 2-M-C3:1 | -2,08 | 0,0372 |  | iso-C15:0 | -2,41 | 0,0160 |  | C8 | 2,31 | 0,0207 |
| 3-M-C4:1 | -2,27 | 0,0234 |  | C9 | -2,60 | 0,0093 |  | 1M-His95 | 2,12 | 0,0344 |
| 2-M-C3 | -2,36 | 0,0184 |  | C6 | -2,65 | 0,0081 |  | Gln | 2,09 | 0,0364 |
| Ala | -2,52 | 0,0118 |  | C18:2 | -2,80 | 0,0051 |  | GABA | 2,02 | 0,0436 |
| C4 | -2,64 | 0,0082 |  | C8 | -3,25 | 0,0011 |  | Ile | 1,98 | 0,0481 |
| C5 | -2,79 | 0,0052 |  |  |  |  |  | Ser | -2,04 | 0,0411 |
| Orn | -2,85 | 0,0043 |  |  |  |  |  | AADP | -2,07 | 0,0385 |
| 2-M-C4:1 | -3,00 | 0,0027 |  |  |  |  |  | C15:0 | -2,28 | 0,0225 |
| Met | -3,04 | 0,0024 |  |  |  |  |  | Gly | -2,35 | 0,0186 |
| Pro | -3,10 | 0,0020 |  |  |  |  |  | Cys-Cys74 | -2,51 | 0,0122 |
| 2-M-C4 | -3,16 | 0,0016 |  |  |  |  |  |  |  |  |
| Tyr | -3,18 | 0,0015 |  |  |  |  |  |  |  |  |
| 3-M-C4 | -3,20 | 0,0014 |  |  |  |  |  |  |  |  |
| Leu | -3,57 | 0,0004 |  |  |  |  |  |  |  |  |
| Val | -3,61 | 0,0003 |  |  |  |  |  |  |  |  |
| Ile | -3,62 | 0,0003 |  |  |  |  |  |  |  |  |
| Phe | -3,70 | 0,0002 |  |  |  |  |  |  |  |  |

HCPC provided three distinct clusters of which each is characterized by respective metabolites from plasma that are either positively or negatively related with each cluster. P-values were derived from t-tests comparing each cluster mean separately with the overall mean and finally transformed to a normal quantile using the v-test transformation by Lebart.
