# Supplementary material for: Acylcarnitine Profiles in Plasma and Tissues of Hyperglycemic NZO Mice Correlate with Metabolite Changes of Human Diabetes
Source: J Diabetes Res. 2018 Apr 26;2018:1864865. doi: 10.1155/2018/1864865 (PMC5944288; doi:10.1155/2018/1864865)
Supplement: Supplementary 2 — Supplemental Table A.2: ANOVA test results and fold changes from plasma-derived phenotypic traits including blood glucose, insulin, TAG, NEFA, and urea to reveal differences among diabetic male NZO mice (Md) and non-/prediabetic male NZO mice (M). [file 1864865.f2.docx]

Supplemental Table A.2. ANOVA test results and fold changes for phenotypic traits among diabetic male NZO mice (Md) and non-/pre-diabetic male NZO mice (M).

| **Metabolite** | **Md - M** |  |
| --- | --- | --- |
| **Plasma** | **p-val** | **Ratio Md:M** |
| Glucose (mg/dl) | 0,006 ** | 2,10 |
| Insulin (ng/ml) | 0,364 | 1,62 |
| Urea (mg/dl) | 0,983 | 1,04 |
| TAG (mg/dl) | 0,975 | 1,22 |
| NEFA (µM) | 0,914 | 1,12 |

Data was first normalized using the negative reciprocals to preserve the right order among values and afterwards tested for significant differences among diabetic (Md) and non-/pre-diabetic (M) male NZO mice using ANOVA. Results include plasma glucose (mg/dl), insulin (ng/ml), urea (mg/dl), TAG (mg/dl) and NEFA (µM). Female NZO mice were included as one group in the inference testing but not depicted in the table. A significance threshold of α < 0.05 was applied.
